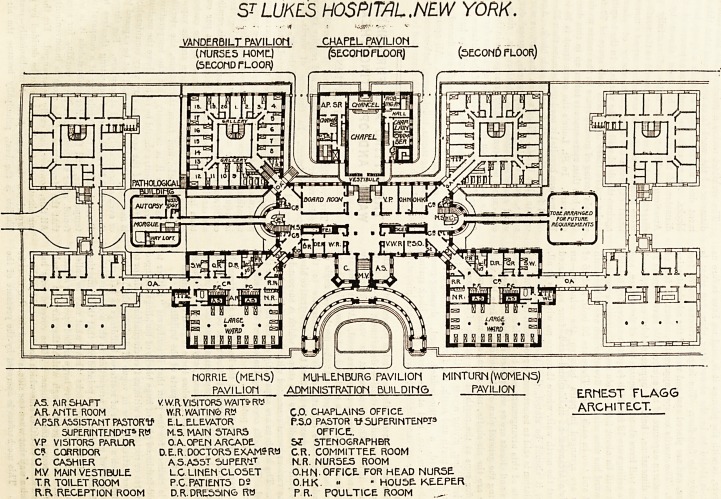# St. Luke's Hospital, New York

**Published:** 1906-10-06

**Authors:** 


### ST. LUKE'S HOSPITAL, NEW YORK.

This hospital stands on an oblong plot of land five hundred and fifty feet long and two hundred feet wide, and when the building is completed there will be twelve blocks. At present six are built. The plan, or, rather, the disposition of the blocks is a very unusual one, and somewhat reminds us of University College Hospital. It is really an attempt to solve a difficult problem which recurs as often as the necessity arises of erecting a large hospital on a limited ground area; and although such a course is not to be recommended it may be said that the architect of the New York Hospital has thought over the problem and solved it fairly well. Whether it would not be better to have had one long corridor running east and west cutting the plot into two unequal parts, about two-thirds to the south and one-third to the north, is another question; but such an arrangement would have given room for six blocks which, if of four stories, would have accommodated nearly five hundred patients. Our business, however, is with the plan as it is, and we have before us what is stated to be the second-floor plan, but what is probably the first-floor. The large pavilions are 76 feet square, and are placed east and west of the administrative block. The wards occupy, apparently, about 35 feet of the southern exposure of the square. We have to guess at these dimensions, but, if approximately correct, each patient will have about 120 square feet of floor space, and as the ceilings are 18 feet high (a quite unnecessary height) there will be about 2,000 cubic feet of air-space per bed. The ward has nine windows, five of which are in the south front and two in each end. There are 20 beds, and 16 of them are arranged in pairs between the windows—an obsolete arrangement in England—and four are placed against the north wall, where there are no windows at all. Here, it should be stated, that the system of artificial ventilation is said to be extremely good, and, therefore, the defects of this arrangement may be obviated. The bathrooms and closets are as well placed as they could be, considering the general plan of the buildings. In a line with these are the air-shafts, ante-room, and nurses' room. A corridor runs at the north of the section of the block we are describing, giving access to the various parts of the block lying north and south, the former including a two-bedded ward, a single-bedded ward, dressing-room, poultice-room, etc., and at the corner of the block springs the corridor of communication with the administrative department.

The main entrance to the hospital is placed between the blocks, and the administrative department is a little further north. This department is very conveniently placed, and all its arrangements are good. At its ends are the staircases, and at the west end is a passage leading to the pathological department. At the north-west angle of the administrative department is the entrance to the nurses' home, and exactly north of the administrative block is the staircase leading to the chapel, which is surrounded by the various rooms connected with the chaplain's department. The kitchens are apparently on the floor under the chapel. The blocks occupying the corners of the site are intended for private patients.

Our readers will notice that the hospital is an extremely compact one, and the administration must be simple. Any block may be reached almost instantaneously from any other block, and yet, if necessary, any block may be isolated from its fellows. Nevertheless there is good circulation of air around the component parts of the building.

It is claimed for the plan that it conforms "neither to the old-fashioned plan of huge blocks of buildings arranged in squares or rectangles without free circulation of air, or to the other extremes now in vogue of dividing the institution into such a number of scattered buildings as to occupy a vast area of ground in proportion to the accommodation furnished." Further, it is pointed out that the connecting corridors of the various blocks "have what may be called fresh air cut-offs, one or more large arched openings at each side of the passages through which the air can freely circulate around the pavilions. . . The covered way has hinged sashes on either side which are connected by a lever in such a way that when one side is closed the other must be open. This mechanism is operated by the pressure of the wind on the sash, therefore, the sash on the windward side is always closed and the sash on the leeward side is always open." This strikes us as being a very good arrangement, and we do not see why it could not be employed on a corridor of any length. Mr. Flagg says these long corridors found in modern hospitals "are, in fact, simply large tubes through which the air from one part of the institution can pass to another part."

The architect makes a point of the fact that the long diameter of the ward faces south, and that this arrangement does not necessitate so wide a space between the blocks. As regards the southern exposure of the long diameter of the ward, it has been shown by another American architect in a series of carefully prepared diagrams that a ward having its end to the south-east gets more sunshine than with any other aspect; moreover the usual exposure obviates the necessity of placing beds against the dead wall of the wards. In the hospital wards under notice there is a north wall space of 70 feet. As regards saving of ground space in the courts no doubt this is so, but then more space is taken up in this direction by the ward. In St. Luke's the south front is 76 feet long. In a modern hospital it need not be more than 32—a saving of 44 feet in the south front of each block. Again, it is certain that in Mr. Flagg's plan the various ward accessories, such as single-bedded wards, nurses' duty-rooms, ward sculleries, store-rooms, bath-rooms, sinks, closets, etc., cannot be so conveniently arranged as in the other plan.

The construction of the building is, as far as possible, fire-proof, but we think in view of the distance of the main staircase from the wards, there ought to have been fire-escapes on each floor. The wards are floored with wood laid in asphalt on a concrete base. All operating-rooms, kitchen, serving-rooms, toilet-rooms, bath-rooms, and closets are lined with glazed tiles or with enamelled bricks.

## Figures and Tables

**Figure f1:**